# Gradual Diaphragmatic Elevation and Chilaiditi Sign Associated With Phrenic Neuropathy Secondary to Cervical Disc Prolapse

**DOI:** 10.7759/cureus.107510

**Published:** 2026-04-21

**Authors:** Luke Galea, Krista Maria Bonello, Kyra Bartolo, Yanika Farrugia, Martin V Balzan

**Affiliations:** 1 General Medicine, Mater Dei Hospital, Msida, MLT; 2 Internal Medicine, Mater Dei Hospital, Msida, MLT; 3 Respiratory Medicine, Mater Dei Hospital, Msida, MLT

**Keywords:** cervical disc prolapse, chilaiditi sign, chilaiditi syndrome, diaphragmatic elevation, phrenic nerve palsy

## Abstract

We report the case of a 78-year-old male who presented to the emergency department with worsening dyspnoea, cough, and wheezing. Chest imaging revealed the Chilaiditi sign with the presence of a progressively elevated right hemidiaphragm. Chest X-rays taken in 2013, 2017, and 2025 showed a progressive rise in the right hemidiaphragm. Magnetic resonance imaging (MRI) of the cervical spine performed in 2025 revealed cervical spinal cord and/or nerve root compression (C3-C5). These MRI findings, together with the progressive nature of the raised right hemidiaphragm over many years, suggest that the Chilaiditi sign may have been associated with this cervical cord/root compression.

The Chilaiditi sign describes the interposition of the colon between the liver and the diaphragm in the right subphrenic space, possibly resulting from various pathologies such as elevation of the right diaphragm, relaxation or damage to the cruciform ligament of the liver, or increased laxity/mobility of the colon.

This case highlights a novel, previously unreported possibility that there might be an association of the Chilaiditi sign with cervical disc prolapse of C3-C5. Further studies are necessary to support this possible association.

## Introduction

The Chilaiditi sign, first described in 1910 by Greek radiologist Demetrius Chilaiditi [1], refers to the presence of either a temporary or permanent herniation of a hollow organ, such as the colon or small intestine, positioned between the liver and the diaphragm. While typically rare and discovered incidentally, the finding is termed Chilaiditi syndrome when it is accompanied by symptoms such as dyspepsia, nausea, vomiting, abdominal pain, respiratory issues, abdominal distension, constipation, intestinal obstruction, or sub-occlusion.

Hemidiaphragmatic paralysis (HDP) may occur due to compression of the phrenic nerve due to space-occupying lesions (both primary and secondary tumours) in the mediastinum along the course of the nerve. Iatrogenic damage may occur following invasive procedures to the neck and thoracic region and upper gastrointestinal surgery. Neurological conditions include multiple sclerosis, Guillain-Barré syndrome, and myasthenia gravis [2]. Cervical disc prolapse with cord or root compression at C3-C5 has been reported to be associated with diaphragmatic paralysis; however, only a handful of cases have been described [3].

We present a case of an elderly gentleman with an incidental finding of an elevated right hemidiaphragm and Chilaiditi sign, which, on retrospective review of serial imaging, demonstrated a gradual progressive elevation of the right hemidiaphragm associated with cervical disc prolapse with compression of nerve roots on nuclear magnetic resonance imaging (MRI).

## Case presentation

A 78-year-old male presented to the emergency department with a one-week history of worsening dyspnoea on minimal exertion, accompanied by a productive cough. 

He had a past medical history of hypertension (HTN), dyslipidaemia, and cerebrovascular accident (CVA) in 2013 with no residual neurological deficit. The only surgical procedure he had undertaken was a right inguinal hernia repair eight years prior to this presentation. He was an ex-smoker of 80 pack-years, with no history of alcohol abuse. Otherwise, he was independent in all activities of daily living.

Before admission, he had started self-administering increased doses of salbutamol inhaler as needed. He denied fever, chest pain, nausea, vomiting, decreased appetite, or weight loss.

On examination, he was found to be tachycardic with a heart rate of 106 beats per minute and had a blood pressure of 158/87 mmHg. On auscultation of the chest, he was found to have bilateral expiratory wheeze. He was neurologically intact with no weakness of either right or left upper limb. Arterial blood gas levels revealed a pO_2_ of 70.6 mmHg, pCO_2_ of 44.4 mmHg (normal <45 mmHg), pH of 7.36, and HCO_3_^-^ of 24.1 mmol/L.

**Figure 1:**
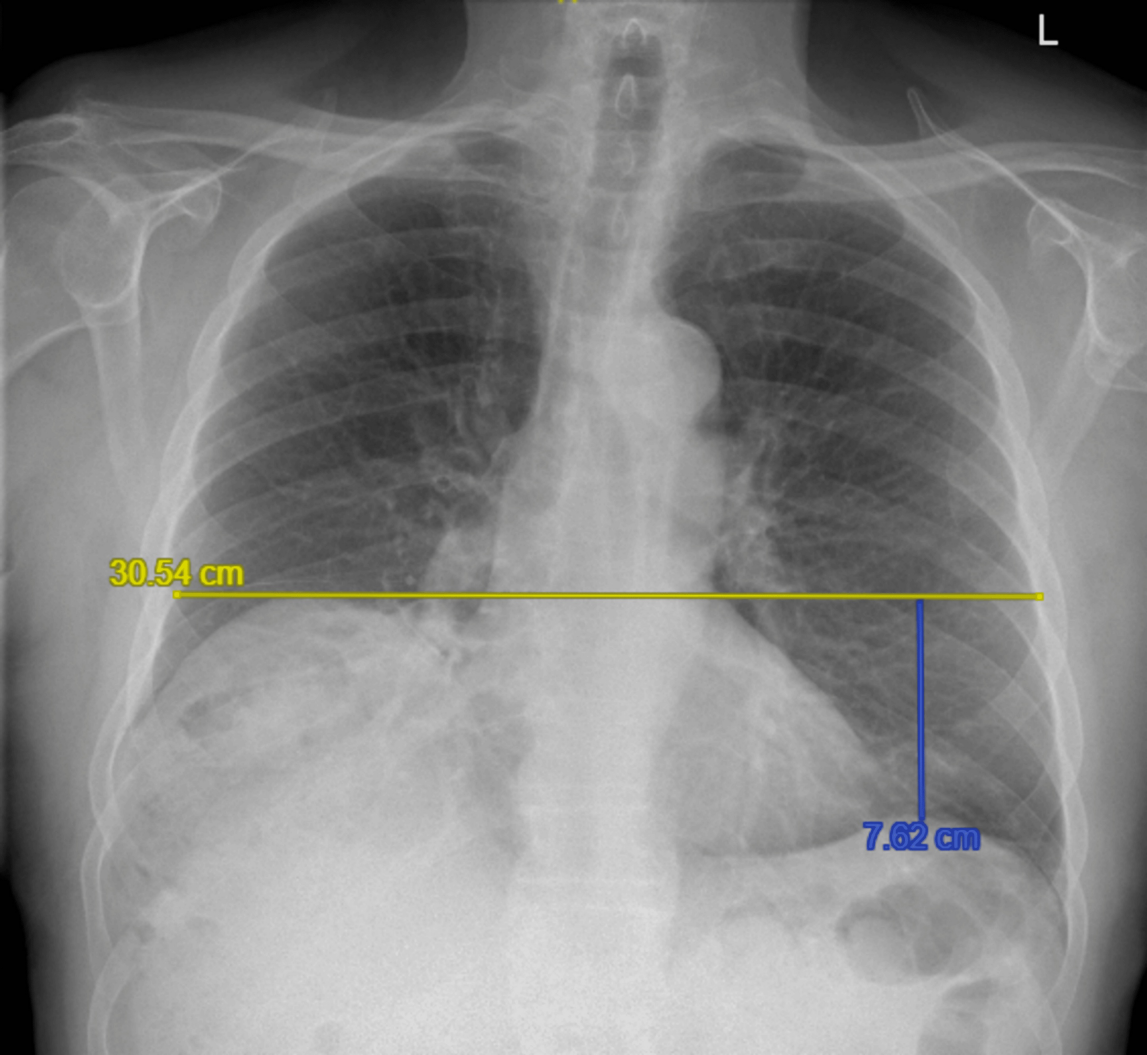
Chest X-ray on presentation in 2025, showing the level of elevated right hemidiaphragm and distance from left hemidiaphragm. Yellow line indicates the higher level of the right hemidiaphragm while the blue line is the height of the right hemidiaphragm in relation to the left hemidiaphragm.

**Figure 2:**
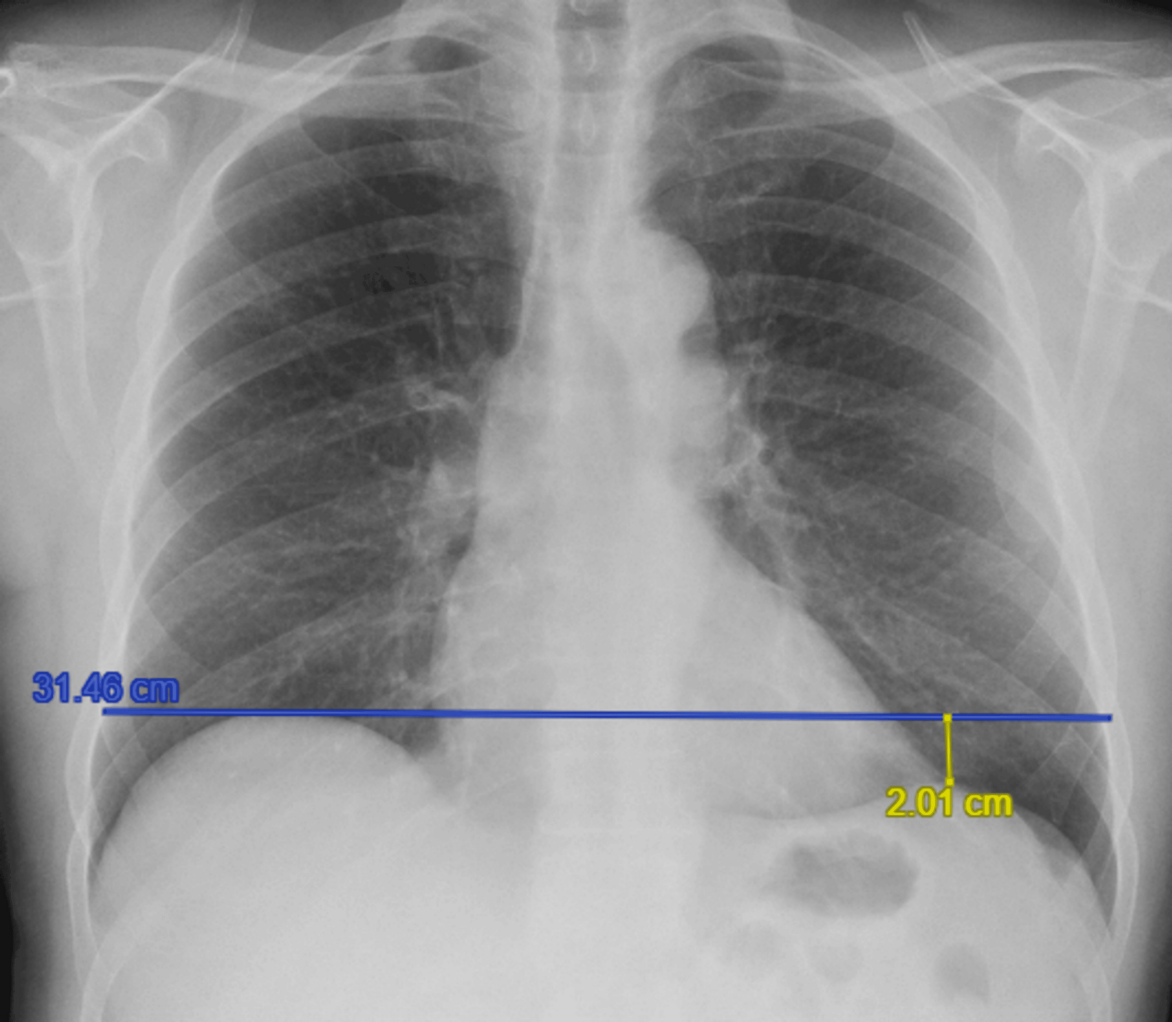
Chest X-ray in 2013, showing normal level of the right hemidiaphragm (blue line), when compared to the left hemidiaphragm (yellow line).

**Figure 3:**
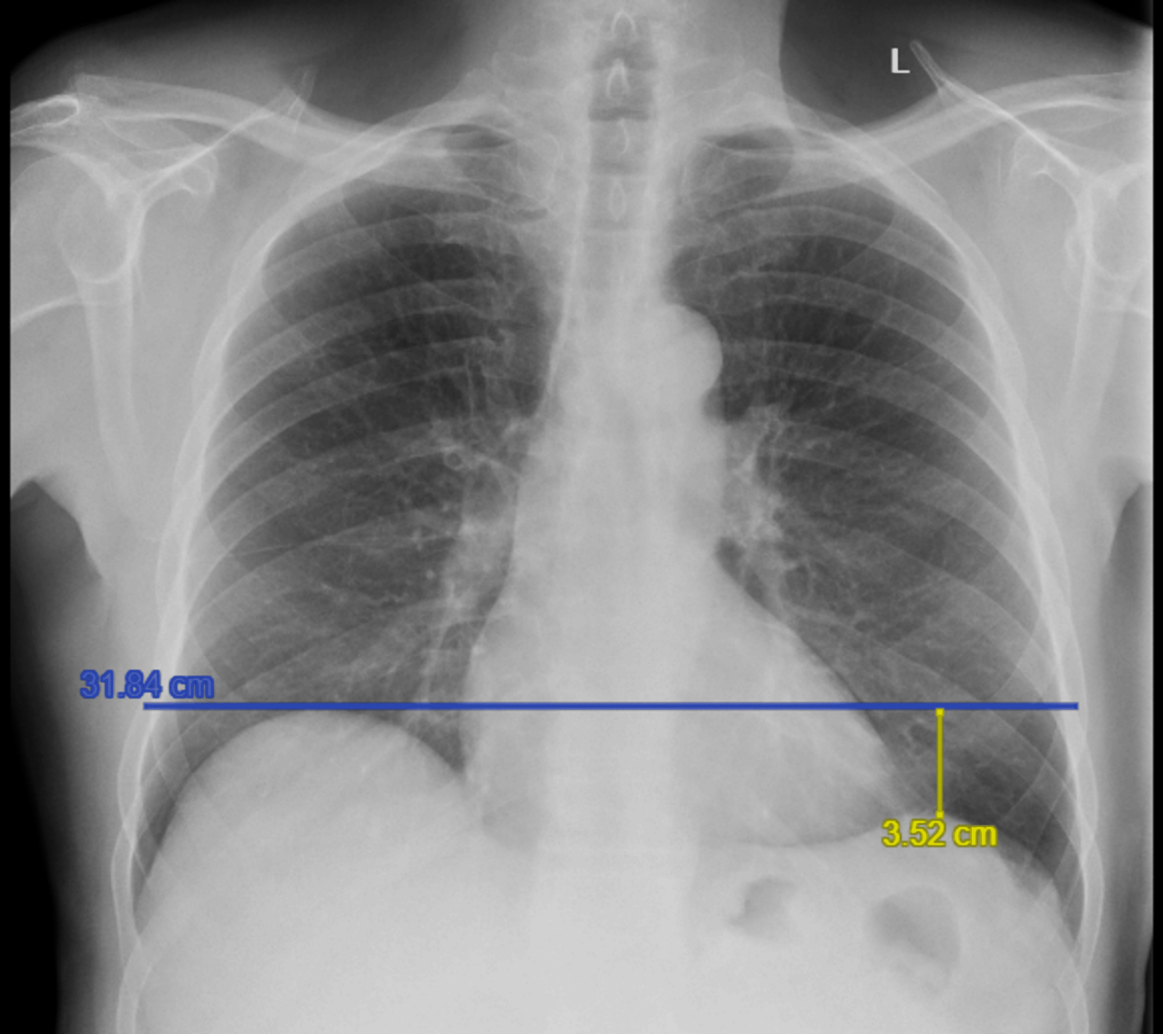
Chest X-ray in 2017, showing mild elevation of the right hemidiaphragm (blue line) when compared to the left hemidiaphragm (yellow line).

On viral screening via nasal swab, the patient was diagnosed with acute bronchitis secondary to co-infection with Influenza B and *Haemophilus influenzae*. Chest radiography demonstrated a clearly elevated right hemidiaphragm by 7.62 cm above the left (Figure 1). Review of prior chest X-rays showed that in 2013, the right hemidiaphragm had been 2.01 cm above the left hemidiaphragm, within the upper limit of normal (<3 cm) (Figure 2) [1]. By 2017, an elevation of 3.52 cm above the left hemidiaphragm was noted (Figure 3).

**Figure 4:**
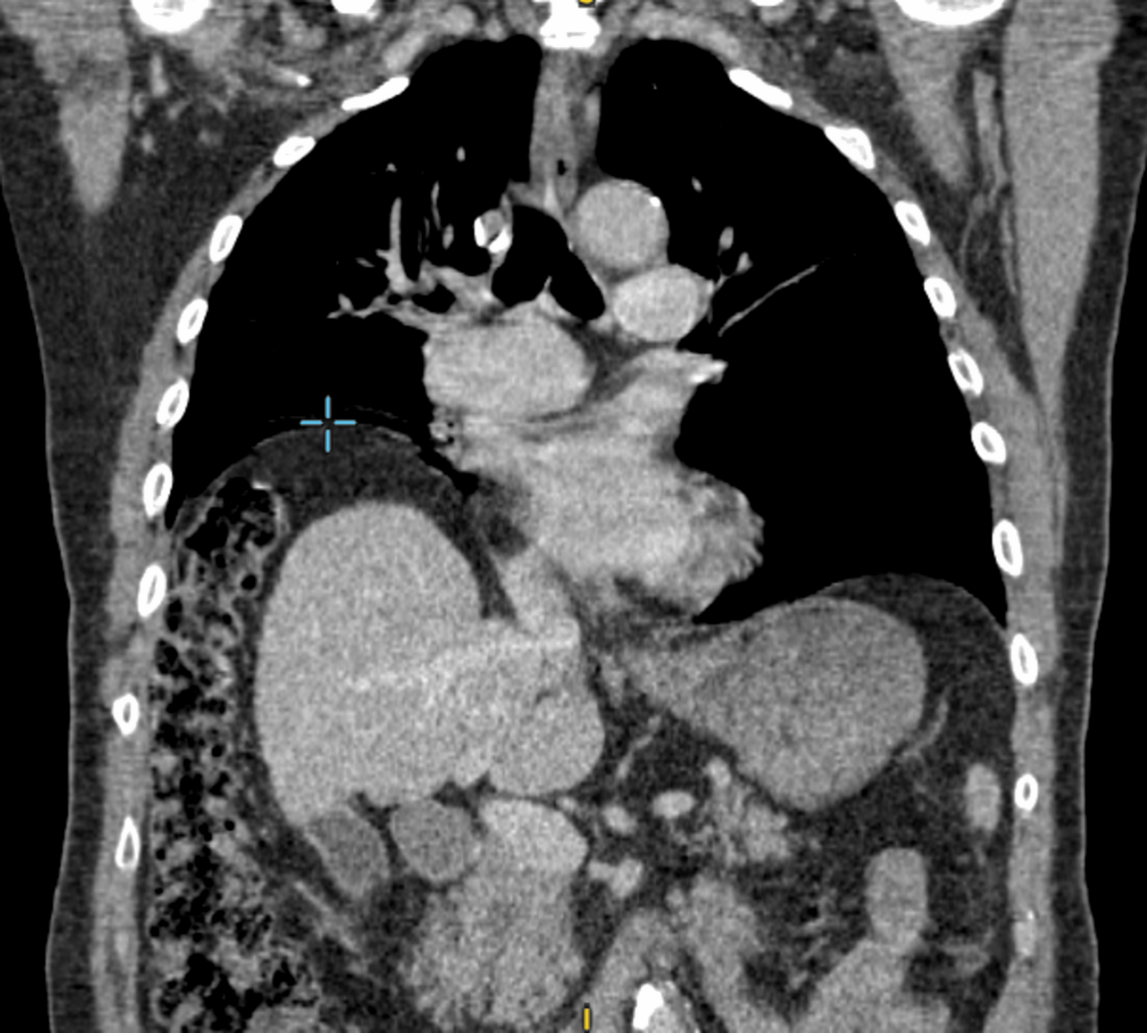
CT thorax coronal view in 2025, showing elevated right hemidiaphragm at the highest point (blue crosshair) with large bowel characterized by typical colonic haustral markings herniating between the liver and the right hemidiaphragm. CT, computed tomography.

A computed tomography (CT) thorax revealed interposition of the large bowel between the liver and diaphragm, consistent with the Chilaiditi sign. Colonic haustral markings are clearly visible, and gas is absent below the diaphragm. There was no evidence of primary or secondary malignancy within the thoracic cavity (Figure 4). 

Following a history of neck pain several months earlier, an MRI of the cervical spine was performed.

**Figure 5:**
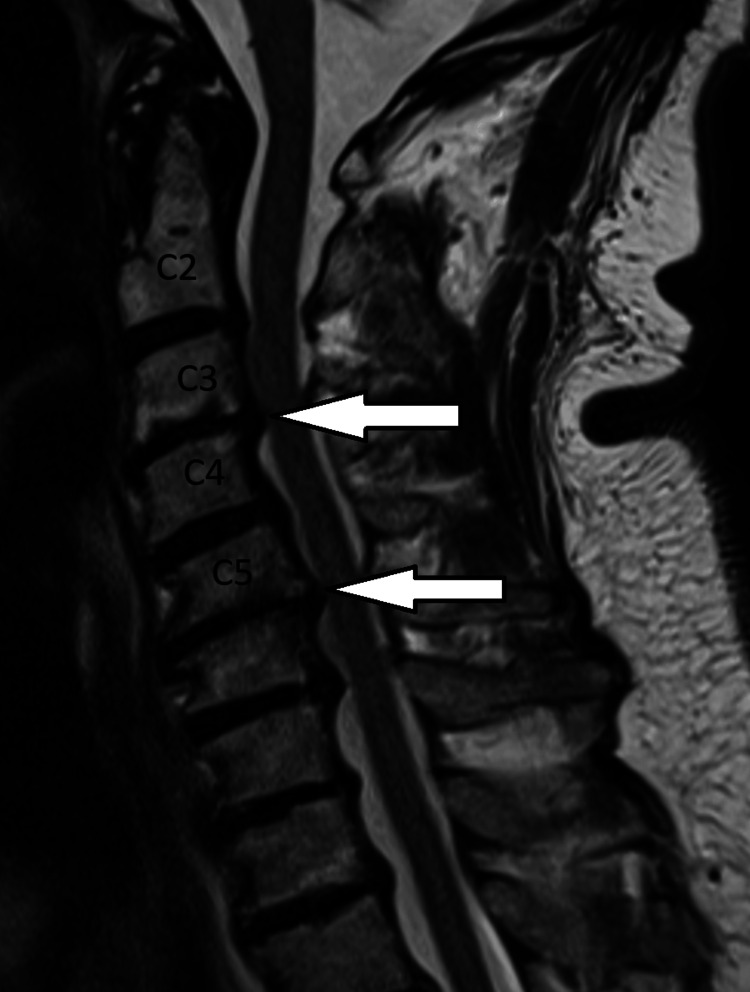
Sagittal T2 sequence MRI spine showing foraminal narrowing, mostly pronounced around C4 and C6 (white arrows). MRI, magnetic resonance imaging.

**Figure 6:**
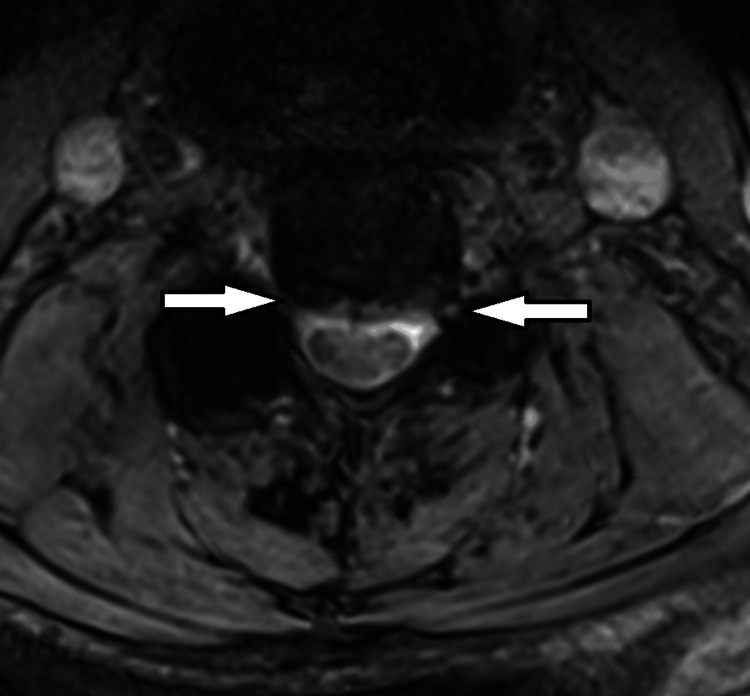
Axial T2 MRI cervical spine showing severe neural foraminal stenosis and canal narrowing at C4 (white arrows). MRI, magnetic resonance imaging.

Figure 5 and Figure 6 show the following:

C2-C3 level: There was a broad-based disc bulge with no cord compression. C3-C4 level: There were Modic type 2 changes of the end plates and bilateral disc osteophyte bars with narrowing of both exiting foramina. C4-C5: Broad-based disc bulge with narrowing of both exiting neural foramina. C5-C6: There was a central broad-based disc protrusion with disc osteophyte bars bilaterally. There was effacement of the thecal sac and moderate narrowing of the neural exit foramina.

The combined CT and MRI findings support the interpretation that, on the balance of probability, phrenic nerve palsy was associated with cervical disc prolapse at C3-C5 and a progressive elevation of the right hemidiaphragm over many years.

**Figure 7:**
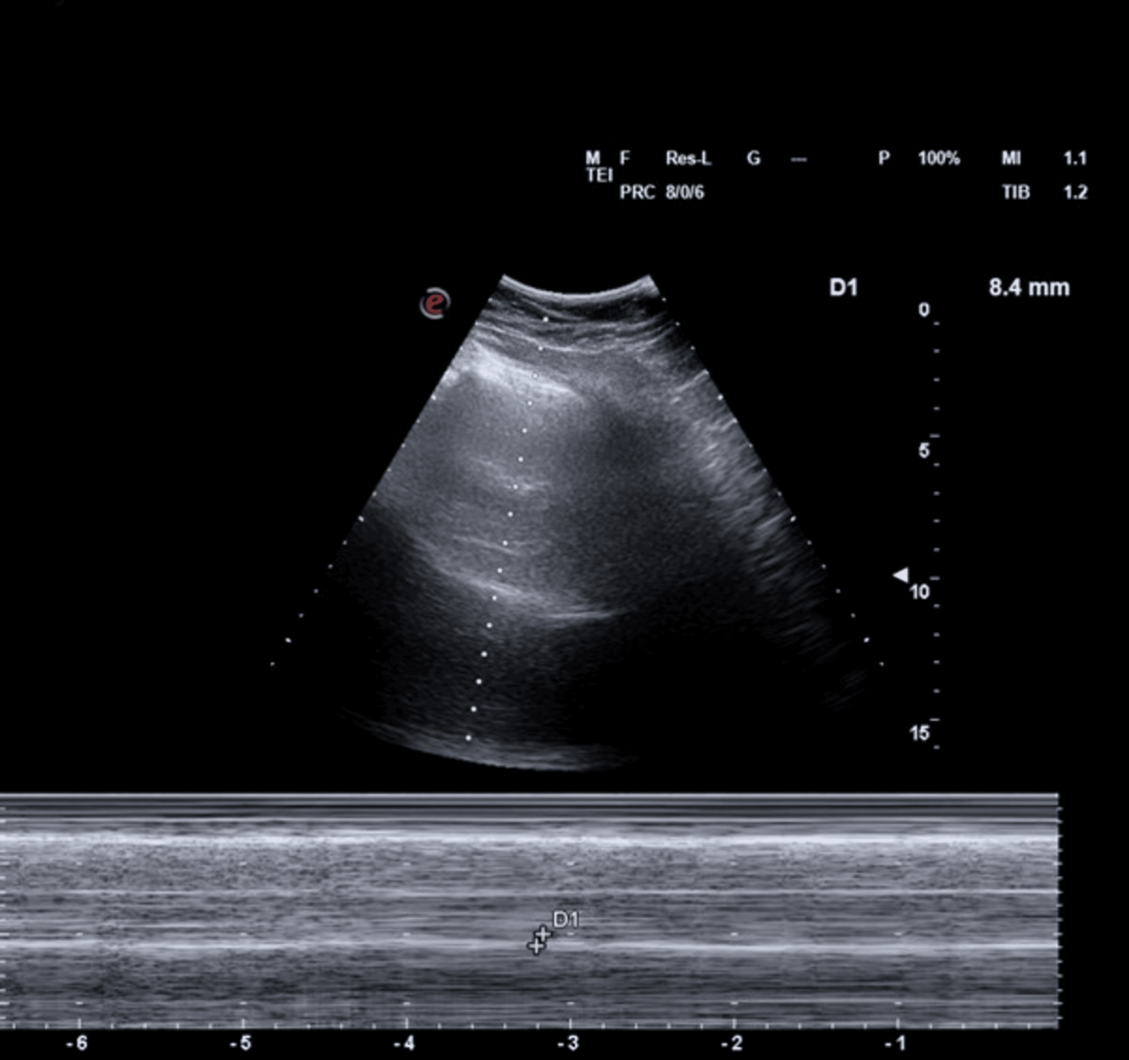
Pleural ultrasound, showing reduced excursion of the right hemidiaphragm.

A pleural ultrasound (US) performed in 2025 further supported this diagnosis, by showing a right hemidiaphragm excursion of 8.4 mm on deep inspiration (Figure 7). This was markedly reduced in relation to the normal excursion of the right hemidiaphragm in male patients, with normal values being between 30 and 70 mm [4]. There was normal hemidiaphragmatic movement on the left.

Lung function tests taken prior to admission had shown a forced expiratory volume in 1 second (FEV_1_)/forced vital capacity (FVC) ratio of 75.9%, FEV_1_ of 1.99 L (82% of predicted), and FVC of 2.68 L (84% of predicted). This revealed a borderline obstructive lung defect, at the lower level of normal (diagnostic threshold for chronic obstructive pulmonary disease, COPD = FEV_1_/FVC of <70%). This was compatible with pre-COPD as defined by the Global Initiative for Chronic Obstructive Lung Disease (GOLD) Guidelines [5] (Table 1).

**Table 1:** Spirometry result one month before admission. Showing pre-COPD as defined by Global Initiative for Chronic Obstructive Lung Disease (GOLD) Guidelines. FEV_1_, forced expiratory volume in 1 second; FVC, forced vital capacity; COPD, chronic obstructive pulmonary disease.

Parameter	Patient value
FEV_1_	1.99 L
FVC	2.68 L
FEV_1_/FVC	75.9%

## Discussion

The Chilaiditi sign refers to the interposition of bowel between the liver and the diaphragm [1]. The underlying cause of the Chilaiditi sign is often unclear and is believed to involve multiple contributing factors. These factors disrupt the normal anatomical relationship between the liver, colon, and diaphragm. Predisposing elements are commonly grouped into three categories: hepatic factors (such as laxity of liver ligaments, cirrhosis, hepatic atrophy, and ascites), intestinal factors (including megacolon, excessive intestinal gas, and abnormal colonic motility), and diaphragmatic factors (such as thinning of the diaphragm, phrenic nerve injury, or changes in intrathoracic pressure, as seen in emphysema). Additional contributing factors may include chronic constipation, prior abdominal surgeries, obesity, and aerophagia [6-8].

Diagnosis can be confirmed by plain radiography; however, CT abdomen provides a clear delineation of bowel position and excludes pneumoperitoneum [8]. The presence of the Chilaiditi sign is backed up by three main radiological criteria: (1) adequate elevation of the right hemidiaphragm above the liver by the loops of the bowel; (2) distention of the intestine with air inside indicating pseudopneumoperitoneum; and (3) shift of the upper liver margin below the level of the left hemidiaphragm [9,10].

The patient presented with acute type 2 respiratory failure as demonstrated by a pCO_2_ of 44.4 mmHg (upper limit of normal = 45 mmHg). Most probably, this was due to a combination of COPD, as shown by the pulmonary function test, further aggravated by the near-complete paralysis of the right hemidiaphragm as established on US. O'Toole and Kramer estimated that paralysis of the right hemidiaphragm can decrease the FVC by up to 50% when supine [11]. However, Caleffi-Pereira et al. concluded that the loss of power from a paralyzed hemidiaphragm can be partially compensated for by the contralateral hemidiaphragm [12]. It is likely that the bronchial infection also contributed to the symptom of dyspnoea.

Abdominal imaging revealed right-sided bowel interposition consistent with the Chilaiditi sign. The finding was incidental and not associated with gastrointestinal symptoms; therefore, the patient did not meet the criteria for the Chilaiditi syndrome. The Chilaiditi sign remains rare, with a reported global prevalence of 0.025-0.28%, and is more frequently observed in male patients (4:1), consistent with this patient’s demographic profile [13,14].

The key predisposing factor in this case was the patient’s chronic right phrenic nerve palsy, resulting in long-standing elevation of the right hemidiaphragm. Advanced cervical disc prolapse, affecting the C3-C5 roots where the phrenic nerve originates, was considered to be the most likely cause. This is supported by the progressive elevation of the right hemidiaphragm on serial chest radiographs, an MRI demonstrating multilevel degenerative cervical spine disease with foraminal narrowing, and markedly reduced diaphragmatic excursion on US [1]. These findings suggest chronic and progressive diaphragmatic dysfunction rather than an acute episode. Furthermore, diaphragmatic excursion on US was only 8.4 mm (normal value 30-70 mm) [4]. Although the patient presented with acute respiratory failure, this was more likely precipitated by an intercurrent chest infection superimposed on pre-existing diaphragmatic weakness.

The chronic elevation of the hemidiaphragm creates an enlarged subdiaphragmatic potential space, in which colonic segments can abnormally migrate. One can speculate that a lax suspensory hepatic ligament may have further facilitated this displacement [15]. Furthermore, when the diaphragm loses its normal hemispheric shape, there is a change in the mechanical forces through the muscle and the ligaments. A flattened diaphragm will result in an altered force pattern, reducing the stabilizing tension on the hepatic flexure and making it more vulnerable to displacement. This combination of muscular dysfunction, ligamentous laxity, and loss of diaphragm shape provides a possible plausible explanation for the development of the Chilaiditi sign in this patient.

Differential diagnosis of an elevated right hemidiaphragm

The investigation of right hemidiaphragm elevation warrants a structured approach as it may be secondary to supradiaphragmatic, diaphragmatic, or subdiaphragmatic pathologies [16]. In this case, a CT thorax was performed to exclude supradiaphragmatic causes in the thorax, particularly primary or secondary malignancies along the long course of the phrenic nerve. No pulmonary masses, atelectasis, fibrosis, hypoplasia, or pleural tumour were identified [17]. Likewise, no subdiaphragmatic causes such as hepatic or splenic collections, hepatomegaly, or hepatic malignancy (including hepatocellular carcinoma or metastatic liver disease) were present. There was no previous history of upper abdominal surgery or major trauma. Diaphragmatic hernia and rupture were also excluded by CT imaging.

Based on the exclusion of these possible diagnoses, the most probable hypothesis was that the elevated right hemidiaphragm may have resulted from neurologic dysfunction of the phrenic nerve secondary to cervical disc prolapses at C3-C5 levels on the right side, compressing on the nerve roots and cord, as documented by MRI images. The association of these anatomical abnormalities may not necessarily have been causal, but this remains the most probable pathology responsible for the right diaphragmatic elevation.

## Conclusions

This case highlights a rare possible association between cervical disc prolapse at C3-C5, phrenic nerve dysfunction with raised right hemidiaphragm, and the Chilaiditi sign.

This paper underscores the importance of evaluating diaphragmatic elevation by performing neurological, respiratory, and radiological investigations when the Chilaiditi sign is suspected. Careful clinical evaluation and radiological correlation may be required to rule out alternative causes, ultimately guiding appropriate management. On the other hand, misinterpretation of the Chilaiditi sign as pneumoperitoneum may lead to unnecessary surgical interventions. Failure to identify underlying diaphragmatic weakness may result in a missed underlying diagnosis.

In this patient, recognition of the true aetiology avoided inappropriate surgical referrals and shifted management towards addressing respiratory compromise and underlying cervical disc prolapse. In this case, the Chilaiditi sign was present, while the absence of any gastrointestinal symptoms excluded the Chilaiditi syndrome, suggesting that this was likely to be of a chronic and long-standing nature and considered to be coincidental.

## References

[1] Patel PR, Bechmann S (2023). Elevated hemidiaphragm. StatPearls [Internet].

[2] Darmanin A, Gatt Y, Miruzzi M, Balzan MV (2024). Invasive procedures to the neck and chest and hemidiaphragmatic paralysis: Are they always causally associated?. Cureus.

[3] Fiott DL, Gauci J, Pace Bardon M, Balzan M (2022). Type 2 respiratory failure secondary to left hemidiaphragmatic paralysis. Breathe (Sheff).

[4] Kabil AE, Sobh E, Elsaeed M (2022). Diaphragmatic excursion by ultrasound: Reference values for the normal population; a cross-sectional study in Egypt. Multidiscip Respir Med.

[5] Global Initiative for Chronic Obstructive Lung Disease. (2025 (2025). Global Initiative for Chronic Obstructive Lung Disease. Global Strategy for the Diagnosis, Management, and Prevention of Chronic Obstructive Pulmonary Disease (2025 Report). https://goldcopd.org/2025-gold-report/.

[6] Leong EKF, Lee SKF, Maduka I (2023). Ogilvie syndrome with bilateral Chilaiditi sign. Indian J Surg.

[7] Caicedo L, Wasuwanich P, Rivera A, Lopez MS, Karnsakul W (2021). Chilaiditi syndrome in pediatric patients - Symptomatic hepatodiaphragmatic interposition of colon: A case report and review of literature. World J Clin Pediatr.

[8] Farkas R, Moalem J, Hammond J (2008). Chilaiditi's sign in a blunt trauma patient: A case report and review of the literature. J Trauma.

[9] Sarkar S, Ramala SR (2024). Chilaiditi sign: A rare radiographic encounter and diagnostic exploration. Cureus.

[10] Lekkas CN, Lentino W (1978). Symptom-producing interposition of the colon. Clinical syndrome in mentally deficient adults. JAMA.

[11] O'Toole SM, Kramer J (2023). Unilateral diaphragmatic paralysis. StatPearls [Internet].

[12] Caleffi-Pereira M, Pletsch-Assunção R, Cardenas LZ (2018). Unilateral diaphragm paralysis: A dysfunction restricted not just to one hemidiaphragm. BMC Pulm Med.

[13] Moaven O, Hodin RA (2012). Chilaiditi syndrome: A rare entity with important differential diagnoses. Gastroenterol Hepatol (N Y).

[14] Weng WH, Liu DR, Feng CC, Que RS (2014). Colonic interposition between the liver and left diaphragm - Management of Chilaiditi syndrome: A case report and literature review. Oncol Lett.

[15] Moore JC, Thompson J, Eckel RM (2012). Chilaiditi sign and syndrome: An unusual case and review of the literature. J Emerg Med.

[16] Shah SS, Chaurasia D, Katuwal D (2022). Dilated sigmoid colon with Chilaiditi's sign mimicking diaphragmatic hernia: A case report. Int J Surg Case Rep.

[17] Kokatnur L, Rudrappa M (2018). Diaphragmatic palsy. Diseases.

